# High burden of hepatitis B infection in Northern Uganda: results of a population-based survey

**DOI:** 10.1186/1471-2458-13-727

**Published:** 2013-08-07

**Authors:** Emmanuel Ochola, Ponsiano Ocama, Christopher G Orach, Ziadah K Nankinga, Joan N Kalyango, Willi McFarland, Charles Karamagi

**Affiliations:** 1 Clinical epidemiology unit, School of Medicine, Makerere University College of Health Sciences, Kampala, Uganda; 2 St. Mary’s Hospital Lacor, Gulu, Uganda; 3 Department of Medicine, Makerere University College of Health Sciences, Kampala, Uganda; 4 School of Public Health, Makerere University College of Health Sciences, Kampala, Uganda; 5 University of California, San Francisco, USA

## Abstract

**Background:**

Worldwide 2 billion people are exposed to hepatitis B infection, 350 million have chronic infection, 65 million in sub-Saharan Africa. Uganda is highly endemic with 10% national prevalence of hepatitis B infection, rates varying across the country from 4% in the southwest and 25% in the Northeast. Childhood vaccination was rolled out in 2002, the effect of which on the burden of hepatitis B has not been examined. We determined the prevalence and risk factors for hepatitis B infection in the Northern Uganda Municipality of Gulu.

**Methods:**

We carried out a cross-sectional, population-based survey. The study population included those found at home at the time of recruitment. Data on demographics, wealth index, cultural and behavioral factors, vaccination and health education on hepatitis B were collected. Hepatitis B infection (Hepatitis B surface antigen positive) and lifetime exposure (anti-hepatitis B core antibody positive) were measured. Analysis was done in 2 age groups, 1–14 years, 14 years and more. Associations between predictors and HBV infection were assessed.

**Results:**

Information on 790 respondents were analyzed. Overall, 139/790 (17.6%) had hepatitis B infection and 572/790 (72.4%) lifetime exposure. In the younger age group 16/73 (21.9%) had hepatitis B infection and 35/73 (48%) lifetime exposure. Increasing wealth was protective for infection (OR 0.46 per quartile, 95% CI=0.26-0.82, p=0.009), while older age was protective for lifetime exposure (OR 2.70 per age group, 95% CI 1.03-7.07, p=0.043). In the older age group, overall hepatitis B infection was seen in 123/717 (17.2%) and lifetime exposure in 537/717 (74.9%). The female sex (OR 0.63, 95% CI=0.42-0.98, p=0.032) and increasing age (OR 0.76 per age group, 95% CI=0.64-0.91, p=0.003) were factors associated with infection. For lifetime exposure, increasing number of lifetime sexual partners was a risk factor (OR 1.19 per partner category, 95% CI=1.04-1.38, p=0.012).

**Conclusions:**

We found a high prevalence of hepatitis B infection and lifetime exposures to hepatitis B in this northern Uganda Municipality. Targeted vaccination of susceptible adults and improving existing childhood vaccinations and provision of treatment for those with infection will play roles in reducing the high prevalence rates seen in the population.

## Background

Hepatitis B viral infection (HBV) causes significant global burden of disease with 2 billion people exposed to the virus, more than 350 million of whom are chronic carriers. It is a cause of more than 600,000 deaths annually. Africa shares 25% of the total HBV burden, with 65 million chronic carriers [1,2]. Infection is highly endemic in Uganda with a national prevalence estimate of 10% in a study carried out in 2004. The in-country distribution of the virus however varies from region to region. The highest prevalence is found in the Northern part of the country ranging from 19% in North west to 25% in the North east [3]. The explanation for this variation in distribution is not well established.

Hepatitis B transmission occurs through blood and blood product exposures. While sexual and needle stick exposures are common modes of transmission in the low endemic areas, transmission in high endemic regions tends to occur in early childhood either perinatally or through child-to-child horizontal methods [2]. Scarifications, shown to be an important mode of HBV transmission in South Africa [4] is also a common practice in northern Uganda but its contribution to transmission of infectious diseases is not studied [5,6].

Control of hepatitis B is done through immunization. Where this has been done the prevalence of infection and chronic liver diseases have been significantly reduced [7]. The Uganda National Expanded Program on Immunizations (UNEPI) successfully helped scale-up childhood immunizations including hepatitis B which was included in the program in the year 2002. However the program strategy incorporates the hepatitis B vaccine into a combination vaccine whose first dose is administered at 6 weeks of age. This was a strategy of World Health Organization and is still being used in many African Countries. However, this delay both limits the efficacy of the vaccine in the prevention of vertical transmission and allows for the potential transmission of hepatitis B through close contacts in the first weeks of life.

Over the last 25 years Northern Uganda has been affected by war and most of the population (including those in the studied Municipality) was sequestered in Internally Displaced People’s (IDP) camps. The conditions in the camps were difficult, with deterioration of health indicators in the camps as was reported in the WHO-Uganda report 2004 [8].

This war has come to an end and most of the camps have been dismantled and a number of people from various parts of the country have flocked into the region seeking for business or employments. We conducted a study to determine the burden of HBV infection and associated factors in Gulu Municipality after the scale up of the hepatitis B vaccination in 2002. Gulu municipality is the main town in Gulu district, Northern Uganda.

## Methods

During the months of January and February 2010 we carried out a cross-sectional community based HBV survey in Gulu Municipality, Northern Uganda. This Municipality has four sub-counties each of which has 4 parishes (making a total of 16 parishes) and 60 villages and the total population in the Municipality is 356,000 according to the population census carried out in the year 2000.

During this study, we selected 8 parishes by probability sampling proportionate to size forming clusters from which 100 households from each of the parishes were selected by systematic random sampling. The households were numbered consecutively. From each household, one member among those who were available at home at the time of the survey was selected by simple random sampling. This was done using papers one of which had a number, the one who picked the number was selected.

Persons were eligible for inclusion into the study if they were aged one year and above, were residents of Gulu Municipality that had been living there for six months or more, were available at home on the day of the survey, and provided informed consent to participate in the study. Informed consent for participants that were aged less than 18 years was provided by the parents or guardians. In addition, children aged 8–17 years provided assent before enrollment into the study.

For all participants interviewer administered questionnaires collecting information on demographic factors [age, gender, marital status, occupation, religion, tribe, and wealth index was computed from ownership of household assets], cultural and behavioral factors [scarification practice, touching of the dead, sharing of towels, sharing of sweets, number of lifetime sexual partners and family history of liver disease] and preventive factors [vaccination against hepatitis B and health education about HBV] was conducted. For adolescents in particular, they answered the questions themselves, but could easily refer to their caretakers when they needed to. For younger children parents provided the answers.

Research assistants, who were nurses, administered the questionnaire and collected 5-8mls of blood from participants. Samples were stored at -2°C to -8°C in the field, and transported to Lacor hospital laboratory within 24 hours, centrifuged and sera extracted. These were used for detecting hepatitis B surface antigen (HBsAg), a marker of HBV infection, and hepatitis B core antibody (anti-HBcAb), a marker of lifetime exposure.

Ethical approval was obtained from School of Medicine research and ethics committee at Makerere University College of Health Sciences and Uganda National Council of Sciences and Technology (UNCST). The study was conducted in accordance with the Helsinki Declaration.

### Serological assay

Serological testing was done in Lacor hospital main laboratory. Hepatitis B surface antigen was determined using HEXAGON HBsAg (HUMAN GmbH, Wiesbaden, Germany). This is an immunochromatographic 1-step test for Hepatitis B surface antigen with results reported as positive or negative. Meanwhile total anti-HBcAb status was assessed using automated ELISA machine (ELYSIS UNO, HUMAN GmbH, Wiesbaden, Germany).

### Data management and analysis

Laboratory results were merged with questionnaire data by unique identifiers. Data was checked for completeness and consistency, partially double entered into Epidata version 3.1 and exported to STATA version 9 for analysis.

We separated the analysis of participants into two categories defined by age 1–14 and those above, because of the confounding or interacting effects of age on most of the variables. Prevalence of HBV infection and lifetime exposure were determined across the age strata as the proportion of tested participants’ specimens that were found to be positive for HBsAg and antiHBc in the studied population respectively. Persons who have HBV infection have both markers, while those who have eliminated the infection have only the markers of lifetime exposure. Associations between predictors and HBV infection and lifetime exposures were assessed using chi square tests, followed by logistic regression analysis producing odds ratios with their 95% confidence intervals. Variables were included in multivariate analysis if they had p-values less than 0.2 or if they had theoretical importance (i.e., reported in the literature as predictors of hepatitis B infection). The latter consideration led to examining family history of liver disease, vaccination history, education on HBV infection, and for older persons scarification and number of sexual partners. A p-value of < 0.05 was significant.

## Results

During the study period, a total of 804 participants from eight parishes in four sub-counties in Gulu Municipality were interviewed. This number slightly exceeded the expected 800 participants because there was misnumbering of the households which led to a repetition of 4 numbers. Two participants withdrew consent before blood draws. Blood samples were drawn from the remaining 802 participants and analyzed for hepatitis B virus markers; 790 had adequate samples for analysis.

Demographic characteristics of the participants are displayed in Table 1. Of 790 participants most (566/790, 71.7%) were of female gender, of age ranging from 1 year to over 45 years. Most participants (496/790, 62.6%) were married, mainly Catholic (615/790, 77.8%) by religion and Acholi (703/790, 89%) by tribe.

**Table 1:** Demographic characteristics of participants of hepatitis B household survey, Gulu, Uganda, 2010 (N=790)

**Variable**	**N**	**Percent**
Sex		
Male	224	28.4
Female	566	71.7
Age group in years		
1 – 14	73	9.2
15 – 24	184	23.3
25 – 34	203	25.7
35 – 44	141	17.9
45+	189	23.9
Marital status		
Single	178	22.5
Married	496	62.6
Divorced/separated	50	6.3
Widowed	66	8.4
Education		
None	137	17.3
Primary	392	49.6
Secondary	220	27.9
Tertiary	41	5.2
Religion		
Catholic	615	77.8
Protestant	104	13.2
Muslim	52	6.6
Pentecostal	19	2.4
Tribe		
Acholi	703	89.0
Lango	15	1.9
Alur	22	2.8
Other*	50	6.3
Occupation		
Healthcare worker	15	1.9
Armed/ security	45	5.7
Others**	730	92.4

* Other tribes-Baganda, Bateso, Banyankole, Congolese and Karimojong.

** Occupation other than health worker or armed services.

### Overall prevalence of chronic HBV infection and lifetime exposure to HBV infection

Overall hepatitis B infection was found in 139/790 participants (17.6%, CI 14.9-20.3) while that of lifetime exposure to infection was in 572/790 (72.4%, CI 69.3-75.5).

### Subgroup analysis for HBV infection and lifetime exposures to HBV infection

#### Participants 1–14 years old

Overall HBsAg in this age group was found in 16/73 (21.9%) (Table 2). On evaluation of associated factors in this age group, only family wealth status was significant, showing protective effect of increasing wealth (OR 0.46 per quartile, 95% CI=0.26-0.82, p=0.009). Lifetime exposure within this age group was seen in 35/73 (48%) and older age was associated with exposure for 11–14 year olds compared to 10 years or under (OR 2.70, 95% CI 1.03-7.07, p=0.043).

**Table 2:** Prevalence of hepatitis B infection and lifetime exposure, children 1–14 years, Gulu, Uganda, 2010 (N=73)

	**Hepatitis B infection (HBsAg positive)**				**Lifetime exposure (Anti-HBcAb positive)**			
**Variable (n)**	**N positive**	**Percent**	**Crude odds ratio (95% CI)**	**p-value**	**N positive**	**Percent**	**Crude odds ratio (95% CI)**	**p-value**
**Overall (73)**	16	21.9	--	--	35	48.0	**--**	**--**
**Sex**								
Male (35)	7	20.0	Ref.	0.704	14	40.0	Ref.	0.194
Female (38)	9	23.7	1.24 (0.41–3.79)		21	55.3	1.85 (0.73–4.70)	
**Age (years)**								
≤10 (32)	8	25.0	Ref.	0.575	11	34.4	Ref.	0.043
11 – 14 (41)	8	19.5	0.73 (0.24–2.21)		24	58.5	2.70 (1.03–7.07)	
**Wealth index**			0.46 (0.26–0.82)*	0.009			0.91 (0.60–1.41)*	0.681
Lowest quartile (11)	5	45.5			6	54.6		
Second quartile (18)	5	27.8			9	50.0		
Third quartile (21)	5	23.8			9	42.9		
Highest quartile (23)	1	4.4			11	47.8		
**Scarification (44)**	12	27.3	2.34 (0.67–8.15)	0.181	20	45.5	0.78 (0.30–1.99)	0.600
**Family history, liver disease** (8)	2	25.0	0.73 (0.15–3.43)	0.697	5	62.5	0.44 (0.104–1.86)	0.265
**Vaccinated against HBV** (10)	2	20.0	1.14 (0.22–6.01)	0.875	3	30.0	2.41 (0.57–10.17)	0.231
**Educated on HBV infection** (3)	1	33.3	0.55 (0.05–6.43)	0.630	2	66.7	0.45 (0.04–5.15)	0.518

*Linear model, odds ratio per quartile increase in wealth index.

#### Par*ticipants 15 years and older*

Overall HBsAg was positive in 123/717 (17.2%) (Table 3). In bivariate analysis factors that showed a significant association for infection included female sex (OR 0.63, 95% CI=0.42-0.98, p=0.032) and younger age (OR 0.76 per age category, 95% CI=0.64-0.91, p=0.003). The other factor that showed border line significance was wealth index. In a multivariate model that included all the above factors, female sex (OR 0.61, 95% CI=0.40-0.93, p=0.021) and age (OR 0.76 per group, 95% CI=0.63-0.90, p=0.002) remained significant correlates for infection.

**Table 3:** Prevalence of hepatitis B infection and lifetime exposure, persons 15 years and above, Gulu, Uganda, 2010 (N=717)

	**Hepatitis B infection (HBsAg positive)**				**Lifetime exposure (Anti-HBcAb positive)**			
**Variable**	**N positive**	**Percent**	**Crude odds ratio (95% CI)**	**p-value**	**N positive**	**Percent**	**Crude odds ratio (95% CI)**	**p-value**
**Overall (717)**	**123**	**17.2**	**--**	**--**	**537**	**74.9**	**--**	**--**
**Sex**								
Male (189)	42	22.2	Ref	0.032	139	73.5	Ref	
Female (528)	81	15.3	0.63 (0.42–0.98)		398	75.4	1.10 (0.75–1.61)	0.618
**Age**			0.76 (0.64–0.91)*	0.003			1.18 (1.01–1.37)*	0.034
15 – 24 (184)	44	23.9			129	70.1		
25 – 34 (203)	36	17.7			149	73.4		
35 – 44 (141	19	13.5			110	78.0		
45+ (189)	24	12.7			149	78.8		
**Religion**								
Catholic (556)	95	17.1	1	0.594	424	76.3	1	0.666
Protestant (93)	18	19.4	1.16 (0.67–2.04)	0.302	69	74.2	0.90 (0.54–1.48)	0.074
Pentecostal/other (19)	5	26.3	1.73 (0.61–4.93)	0.220	11	57.9	0.43 (0.17–1.09)	0.167
Muslim (49)	5	10.2	0.55 (0.21–1.43)		33	67.4	0.64 (0.34–1.20)	
**Wealth index**			1.17 (0.98–1.39)*	0.080			0.88 (0.76–1.03)	0.108
Lowest quartile (186)	24	12.9			144	77.2		
Second quartile (180)	27	15.0			138	76.7		
Third quartile (177)	42	23.7			133	75.1		
Highest quartile (174)	30	17.2			122	70.1		
**Scarification** (381)	61	16.0	0.84 (0.57–1.24)	0.387	288	74.9	1.08 (0.77–1.52)	0.648
**Vaccinated against HBV** (12)	1	8.3	2.30 (0.29–17.00)	0.427	8	66.7	1.50 (0.45–5.05)	0.51
**Educated on HBV infection** (64)	13	20.3	0.79 (0.42–1.51)	0.483	44	68.8	1.40 (0.80–2.45)	0.237
**Number of lifetime sex partners**			1.08 (0.92–1.27)*	0.323			1.19 (1.04–1.38)*	0.014
None (39)	7	18.0			20	51.3		
1 (229)	35	15.3			166	72.5		
2 (200)	32	16.0			160	80.0		
3 (99)	21	21.2			72	72.7		
4+ (150)	28	18.7			119	79.3		

*Linear model, odds ratio per increase in category.

Final multivariate model for HBV infection included female sex (adjusted OR 0.61, 95% CI 0.40–0.93), p-value 0.021) and age (adjusted OR per group 0.76, 95% CI 0.63–0.90, p-value 0.002).

Final multivariate model for lifetime infection included number of lifetime sex partners (adjusted OR 1.20 per partner category, 95% CI 1.04–1.38, p-value 0.012).

Life time exposure for hepatitis B was found in 537/717 (74.9%) (Table 3). In bivariate analysis, factors significantly associated with exposure were age (OR 1.18 per group, 95% CI=1.01-1.37, p=0.034), and number of life-time sexual partners (OR 1.19, 95% CI=1.04-1.38, p=0.014). Other borderline significant factor was the Pentecostal / other religion category. On adjusting for all the above factors in multivariate analysis only number of lifetime sexual partners (adjusted OR 1.20 per partner category, 95% CI=1.04-1.38, p=0.012) was significant correlate of lifetime exposures for hepatitis B infection.

## Discussion

In this cross-sectional population-based study we have shown that Gulu Municipality in Northern Uganda has a high prevalence of hepatitis B virus infection of 17.6% and a lifetime exposure of 72.4% in the general population. Rates of infection and lifetime exposure in children was 21.9% and 48%, respectively, while in adults the Figures are 17.2% and 74.9%, respectively. A previous national sero-survey (2004) similarly showed high rates of chronic infection and lifetime exposures of 20% and 90%, respectively, in this part of the country [3]. This study was carried out at a time when UNICEF was just scaling up vaccination against hepatitis B in the country. The high prevalence rates shown in our study predict massive potential for ongoing hepatitis B transmission, chronic liver disease and liver cancer.

Most epidemiologic studies show transmission of hepatitis B in the areas of high endemicity occur either perinatally from mother to child exposures or horizontally from child to child in early childhood [2,9]. The high infection rate among the children is rather surprising given this study was carried out at a time when early childhood vaccination was entering its first decade in Uganda. Table 2 shows that by 14 years over one half of the children are already exposed to infection at a time when sexual exposure is presumably at the minimum. This, combined with the finding in Table 3 showing exposure of up to 78.8% by age 45 and above means that most of the exposures seem to occur in childhood. This leaves a range of possible mechanisms for continued transmission. In the first place mother to child perinatal transmission could be taking place since mothers are not routinely screened during the antenatal period to determine infection, losing the opportunity for intervention with post exposure prophylaxis to the babies by provision of hepatitis B immune globulin and the first dose of hepatitis B vaccine in the first 24 hours after delivery [10]. In addition, horizontal transmission is likely because of delayed administration of the initial dose of vaccine which is given at 6 weeks post delivery. Infection may also occur early through breast feeding when vaccination is not administered. A recent metanalysis confirmed breast feeding is not a significant mode of transmission of hepatitis B in vaccinated children [9,11] In our study setting we can speculate this transmission may occur within the period before the administration of the vaccine especially if the mother has cracked or exudative nipples. Even where vaccination is given, UNICEF estimated the prevalence of receipt of the third dose of HBV vaccination nationally at about 80% which also suggests some level of incomplete vaccine immunity [12]. With the disruption of peace in the North there are probably a number of babies born in the villages and who are not exposed to vaccination. Another possibility would be transmission in a setting of family clustering, where a number of the persons in the family could be infected. All these factors would possibly increase chances of hepatitis B transmission to a new born baby in Northern Uganda.

Among the older group (more than 14 years) this high prevalence of infection and exposures especially with the exposure increasing with number of sexual partners suggests ongoing transmission probably through sexual intercourse. Indeed increasing age and number of lifetime exposures have been shown to increase risks of hepatitis B transmission in adults [13]. Because of this high prevalence in the adults and potential for transmission, screening of the population to identify those with infection and those exposed could improve decision on control. Population screening may be difficult but in a recent World Health Organization expert panel discussion it was suggested this could be done through already existing facilities as is with HIV infection [14]. Screening for HIV through voluntary counseling and testing (VCT) services and antenatal visits for prevention of mother to child transmission of HIV has been widely used and accepted in Uganda [15,16]. Hepatitis B and HIV share similar modes of transmission and similar services could be used for hepatitis B screening. Identifying the infected and the exposed would provide information on monitoring and treatment for those who are infected, and targeted vaccination of the adults. Capitalizing on these opportunities would eventually slow down the chain of transmission and the prevalence of disease in the community.

Our study had several limitations. We were not able to measure the anti-hepatitis B surface antibody (anti-HBsAb) in those who were exposed, the hepatitis B e antigen (HBeAg) and hepatitis B viral loads (HBV DNA) in those with infection. These would improve information on protective levels of the antibodies in those infected and determine requirement for treatment and transmissibility for those with infection. However, the main purpose at the time was basically to determine the infection and exposures in the community. This would lead to further studies that will assess effects of screening and possibilities of vaccination and treatment. Secondly, there could have been selection bias as males and children were not proportionately selected because the study sampled from people who were available at home on the day of study. In the studied population the male gender is the main income earner in the homes while most women would stay home. Also a number of children were at school, or playing in the neighborhood during the time of sampling and recruitment. This could potentially lead to an underestimation of prevalence among the male population and children. However we used probability sampling to select available members, meaning that all available had equal opportunity of selection and our sample size was robust. Given that we did not have a census either of the study catchment area or of the households at the time of interviewing, creating weights to account for this could not be possible. Also we did not have the data that would enable adjustment for clustering. Thirdly, the study could have failed to detect persons in the window period, underestimating the prevalence rates seen. This is common in most sero-epidemiology studies on hepatitis B in Africa and may minimally affect the prevalence measure [17].

## Conclusion

We found a high prevalence of hepatitis B infection and lifetime exposures to hepatitis B in this Northern Uganda Municipality. Maximizing on the existing childhood vaccination and identification and targeted vaccination of unexposed adults and treatment of the infected will play big roles in reducing this high prevalence rates seen in the population. Hepatitis B now has safe and effective prevention and treatment available – the time is imminent for Uganda to benefit from them.

## Competing interests

The authors declare that they have no competing interests.

## Authors’ contributions

EO and PO participated equally in conception, design and development of the proposal. EO collected the data. Both participated in statistical analysis and drafted the manuscript. CGO, JNK, ZKN and CK proofread and edited the proposal and reviewed the manuscript. WM supervised and performed the final data analysis, read, edited and approved the manuscript before submission. All authors read and approved the final manuscript.

## Pre-publication history

The pre-publication history for this paper can be accessed here:

http://www.biomedcentral.com/1471-2458/13/727/prepub
